# The effects of home-based HIV counseling and testing on HIV/AIDS stigma among individuals and community leaders in western Kenya: Evidence from a cluster-randomized trial^1,2^

**DOI:** 10.1080/09540121.2012.748879

**Published:** 2013-06-09

**Authors:** Corinne Low, Cristian Pop-Eleches, Winnie Rono, Evan Plous, Angeli Kirk, Samson Ndege, Markus Goldstein, Harsha Thirumurthy

**Affiliations:** a Department of Economics, Columbia University, NY, USA; b School of International and Public Affairs, Columbia University, NY, USA; c College of Health Sciences, School of Public Health, Moi University, Eldoret, Kenya; d Department of Agricultural and Resource Economics, University of California, Berkeley, CA, USA; e The World Bank, Washington, DC, USA; f Department of Health Policy and Management, Gillings School of Global Public Health, University of North Carolina at Chapel Hill, Chapel Hill, NC, USA

**Keywords:** HIV/AIDS, stigma, HIV testing, community, randomized trial

## Abstract

HIV counseling and testing services play an important role in HIV treatment and prevention efforts in developing countries. Community-wide testing campaigns to detect HIV earlier may additionally impact community knowledge and beliefs about HIV. We conducted a cluster-randomized evaluation of a home-based HIV testing campaign in western Kenya and evaluated the effects of the campaign on community leaders’ and members’ stigma toward people living with HIV/AIDS. We find that this type of large-scale HIV testing can be implemented successfully in the presence of stigma, perhaps due to its “whole community” approach. The home-based HIV testing intervention resulted in community leaders reporting lower levels of stigma. However, stigma among community members reacted in mixed ways, and there is little evidence that the program affected beliefs about HIV prevalence and prevention.

## Background

HIV/AIDS-related stigma and discrimination have long been recognized as a barrier to the uptake of HIV prevention, care, and treatment services. Stigma and discrimination have the potential to affect the health, economic, social, and emotional outcomes of individuals already in care and treatment and also to limit efforts to effectively deliver HIV prevention and treatment services (UNAIDS, 2007). Uptake of HIV testing and counseling, which is central to the success of both HIV prevention and treatment efforts, can also depend on stigma through several different mechanisms such as prejudice, stereotypes, and discrimination toward people living with HIV/AIDS (Earnshaw & Chaudoir, 2009). For example, anticipation of stigma has been shown to deter pregnant women from accepting HIV testing in Kenya (Turan et al., 2011). A key priority going forward has thus been to develop interventions that can effectively reduce stigma in communities affected by HIV/AIDS. In this regard it is important to learn whether community-based HIV testing programs, which seek to increase people's awareness of their own HIV status, can be successfully implemented even in the presence of HIV/AIDS-related stigma and whether they can eventually reduce levels of stigma.

One such community-based intervention is door-to-door home-based counseling and testing (HBCT) services. HBCT offers several potential advantages over other methods of testing provision and is beginning to be implemented in several countries (Mulogo, Abdulaziz, Guerra, & Baine, 2011; Nuwaha et al., 2012;Vreeman et al., 2010). HBCT may enable earlier diagnosis of HIV among currently infected individuals. A recent evaluation of the HBCT program that we study in Kenya found that patients who were referred to clinics from home-based testing (Wachira, Kimaiyo, Ndege, Mamlin, & Braitstein, 2012) had median CD4 cell counts of 323 cells/mL, versus 217 cells/mL from voluntary counseling and testing (VCT) clinics and 190 cells/mL from provider-initiated testing and counseling. Additionally, HBCT has been shown to be similar in terms of costs per patient compared to other methods of HIV testing such as VCT (Menzies et al., 2009).

By avoiding the need for individuals to be singled out or to single themselves out to seek testing, HBCT may also potentially blunt the impact of stigma on testing uptake. Since all individuals are approached equally for HIV testing, and encouraged to comply as a community, HBCT may be a mode of testing that can be successfully implemented even in the presence of stigma. Furthermore, there is a possibility that by increasing awareness of HIV/AIDS and individual HIV status in the community and by bringing HIV services to the community, HBCT may actively *reduce* HIV/AIDS stigma in addition to more direct prevention and treatment benefits. If many individuals in the community are offered a chance to be tested for HIV, it may be that anticipated stigma – the fear of the consequences of testing HIV-positive – is lessened. Stigma or discrimination from partners, family, and community members may also be reduced if general awareness and acceptance of HIV/AIDS grow as a result of community sensitization and one-on-one discussions with counselors who visit houses.

This paper addresses the following three research questions: First, can HBCT be successfully implemented and achieve high levels of testing uptake in the presence of stigma? Second, what is the impact of HBCT on the levels of perceived community stigma and levels of self-stigma? Third, what is the impact of HBCT, which includes community leader education and mobilization, on community leaders’ knowledge, attitudes, and behaviors with regard to HIV?

## Methodology

### Study setting

This study was conducted in communities served by a partnership between USAID and the Academic Model Providing Access to Healthcare (AMPATH). In January 2009, USAID-AMPATH began an initiative to implement HBCT in western Kenya, among primarily rural households. The HBCT program began with a community sensitization program in which facilitators, usually drawn from the local community, worked with local government officials to explain the program to the community. Next, locally based counselors visited all of the households in the community to provide voluntary HIV counseling and testing to all consenting adults in a given household. These tests and the associated counseling were administered within the household, and couples were encouraged to test together. Individuals who tested positive for HIV were referred to the local treatment facilities administered by AMPATH for appropriate treatment.

### Sampling strategy and survey data

This study was conducted in two divisions (Burnt Forest and Teso) that contained a total of 35 “locations” (Kenyan administrative region). Among these areas, randomization between matched pairs of locations was used to select 18 locations in which HBCT was to be implemented by USAID-AMPATH mid-way through 2009 (intervention locations). The remaining 17 locations (control locations) were selected for deferred implementation of HBCT in late 2011. Baseline and follow-up household surveys were conducted with a representative sample^5^ of 2700 households drawn from an initial census of the 35 locations. The baseline survey was conducted between April and June 2009. Then, HBCT was implemented by USAID-AMPATH in the intervention locations. In the spring of 2011, approximately 18 months after HBCT was implemented in the intervention locations, all households that participated in the baseline survey were revisited for a follow-up survey. Finally, once the follow-up survey was completed, HBCT implementation was expanded to the control locations, concluding in March 2012.

**Appendix Figure 1.**
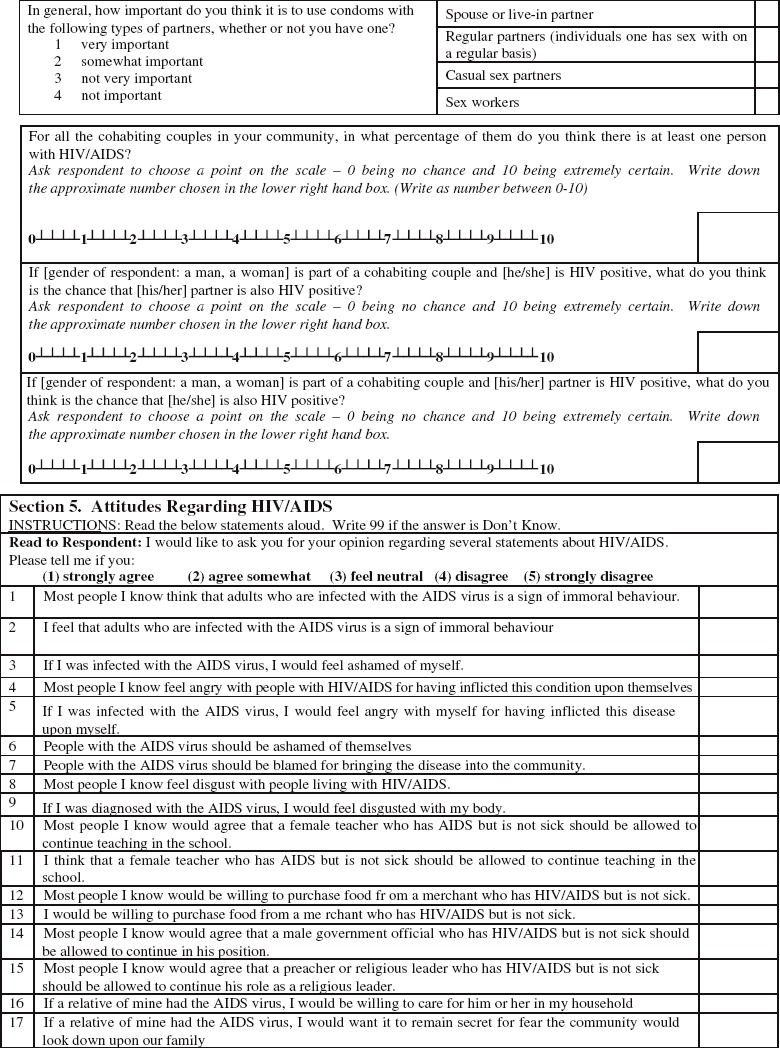
Survey questions on condom importance, HIV knowledge, and stigma beliefs

The data used in this paper came from three sources: baseline and follow-up household surveys, community leader surveys, and administrative data. Household and community leader surveys were conducted in the language of the respondents’ choice, mostly Swahili and Kalenjin in Burnt Forest and Ateso in Teso. The household surveys solicited self-reported information on the respondents’ knowledge about HIV/AIDS, attitudes about HIV/AIDS, and views related to AIDS-related stigma. Stigma can be measured through different scales that measure separate dimensions of stigma (Genberg et al., 2008), such as “perceived community stigma” and “self stigma.” Stigma questions were based on field-tested instruments that have been used by the authors in other studies in Kenya (Thirumurthy, Zivin, & Goldstein, 2008; Goldstein, Zivin, Habyarimana, Pop-Eleches, & Thirumurthy, 2009) and were drawn from several sources including the demographic and health surveys and Genberg et al. (2008). Appendix Figure 1 shows the questions from the survey used in this part of the analysis.

To summarize the stigma questions and reduce the variability in response, we also created five stigma indexes from our 17 stigma questions, averaging component questions (available in Appendix 1).

For the community leader survey, completed at follow-up only, 200 villages in the intervention areas and 200 villages in the control areas were initially targeted, with 313 community leader interviews from all areas completed.^8^ These leaders comprise village elders, religious leaders, political leaders, and women and youth leaders.

Administrative data from USAID-AMPATH were used to calculate testing rates at the village level. To calculate the percent of households tested by village, we assigned a household as “tested” if either the household head or the head's spouse was tested. We then used survey data to compare this result to self-reports indicating how many individuals had ever received an HIV test.

To compare postintervention outcomes between household respondents (or community leaders) in areas assigned to intervention or control, we performed t-tests on the differences between these two groups. For some outcomes, we implemented a regression analysis that includes an intervention indicator variable as well as a number of covariates. All analyses were conducted using Stata version 10.0.

## Results

Table 1 (Panel A) summarizes socioeconomic characteristics of household and community leader respondents: 92% of household respondents had ever been married, their average age was 45 years, 38% were male, and average schooling was 8.6 years. By contrast, community leaders were on average older (49 years), more educated (10.4 years of schooling), and more likely to be male (82%). Table 1 (Panel B) summarizes the main outcome variables and compares the responses of household survey respondents and community leaders.

**Table 1.** Basic details and summary statistics.

	Community leaders					Individuals				
Panel A: Basic characteristics	N	Mean	SD	Min	Max	N	Mean	SD	Min	Max
Male	313	0.8	0.4	0	1	3383	0.4	0.5	0	1
Elected	313	0.5	0.5	0	1					
Age	285	49.4	11.7	20	78	3277	45.0	16.2	3	120
Ever married	313	0.9	0.3	0	1	3383	0.9	0.3	0	1
Completed primary	313	0.8	0.4	0	1	2807	0.5	0.5	0	1
Years of education	281	10.4	3.6	0	19	2803	8.6	3.4	0	22

	Community leaders					Individuals				
Panel B: Beliefs	N	Mean	SD	Min	Max	N	Mean	SD	Min	Max
Beliefs about prevalence/discordance										
% partners where one is +	262	43.6	26.5	0	100	3276	43.5	24.5	0	100
If self +,% partner also +	263	72.1	25.5	10	100	3314	74.6	24.9	0	100
If partner +,% also +	263	72.7	24.9	10	100	3312	75.7	24.4	0	100
Should use condom with …										
Spouse	304	2.7	1.2	1	4	3179	2.4	1.2	1	4
Regular partner	304	3.7	0.7	1	4	3186	3.3	1.0	1	4
Casual partner	304	3.9	0.4	1	4	3188	3.8	0.5	1	4
Sex worker	304	3.9	0.3	1	4	3186	3.9	0.5	1	4
Stigma beliefs										
Most people think HIV immoral	304	3.6	1.7	1	5	3316	3.7	1.5	1	5
Self thinks HIV immoral	304	3.2	1.7	1	5	3335	3.4	1.6	1	5
Would be ashamed if +	301	2.5	1.6	1	5	3335	2.7	1.6	1	5
Most are angry at those with HIV	303	2.6	1.6	1	5	3265	3.0	1.5	1	5
Would be angry at self if +	285	2.5	1.7	1	5	3331	2.7	1.6	1	5
People with HIV should be ashamed	304	2.2	1.5	1	5	3332	2.3	1.4	1	5
People with HIV should be blamed	304	2.0	1.5	1	5	3340	2.4	1.5	1	5
Most people feel disgust	303	2.7	1.6	1	5	3284	2.9	1.5	1	5
Would be disgusted with self	300	2.3	1.6	1	5	3327	2.5	1.5	1	5
Most would think HIV+ teacher OK	303	1.7	1.3	1	5	3311	2.0	1.4	1	5
Self thinks HIV + teacher OK	304	1.5	1.2	1	5	3337	1.8	1.4	1	5
Most would not purchase from + vendor	304	1.8	1.2	1	5	3330	2.2	1.4	1	5
Self would not purchase from + vendor	304	1.6	1.1	1	5	3340	1.9	1.4	1	5
Most think + govt official bad	304	1.5	1.1	1	5	3334	1.9	1.3	1	5
Most think + religious leader bad	305	2.0	1.5	1	5	3333	2.2	1.6	1	5
Self would not care for + person	304	1.2	0.6	1	5	3345	1.4	0.9	1	5
Would want + relative to be secret	304	2.6	1.8	1	5	3339	2.7	1.7	1	5

Note: All statistics are at the time of the follow-up survey.

### Implementing HBCT in the presence of stigma

Table 2 reports uptake of HBCT testing in intervention and control locations, based on administrative data. Testing rates in intervention areas ranged from a minimum of 23% in Okuleu (an urban area where household members are frequently away during the day) to a maximum of 88% in Chuiyat. We found no negative relationship between reported stigma and levels of testing – if anything, higher pre-HBCT stigma communities had *higher* rates of testing. In other words, higher levels of stigma in a community did not interfere with successful implementation.

**Table 2.** Percent of households testing.

Panel A: Percent tested by location				
	Control		Intervention	
Location	% Tested	N	% Tested	N
Amagoro	0.00	59	66.32	95
Angurai	0.00	101	67.44	43
Chemasiri	1.08	93	72.55	51
Chepkero	0.00	29	77.22	79
Chepngoror	0.00	41	87.23	94
Cheptiret	0.00	68	88.37	43
Kamolo	0.00	66	70.83	72
Kamurai	0.00	97	69.23	26
Kapkoi	0.00	61	58.33	48
Kapngetuny	1.56	64	76.47	51
Katakwa	1.03	97	84.81	79
Kesses	0.00	35	79.80	99
Kipsinende	0.00	42	83.33	96
Kocholia	0.00	97	23.36	107
Megun	0.00	60	85.00	40
Olare	0.00	71	62.71	59
Olleinguse	0.00	70	72.06	68
Osajai	0.00	102	68.89	45

Panel B: Relationship between stigma and percent tested in multivariate regression		
	% Tested	% Tested (with controls)
Stigma of most people toward HIV +	0.00308	0.00265
	(0.0295)	(0.0318)
Community's actions toward HIV +	0.0669^***^	0.0716^***^
	(0.0243)	(0.0264)
Self stigma if infected	0.0229	0.0194
	(0.0223)	(0.0236)

Notes: Panel A: Proportion of households who remained in the sample at the time of the follow-up survey who participated in testing via the HBCT campaign. A household was coded as “tested” if one of the adult members of the household tested. Panel B: Standard errors in parentheses;

*** *p*<0.01, **p<0.05, *p<0.1.

Table 3 reports self-reported information from the household survey on whether individuals had ever had an HIV test. In intervention locations, the percentage of people who had ever received an HIV test was 95%, compared to 64% in control locations. In populations in which prior HIV testing rates were relatively lower in control locations, the likelihood of ever having tested for HIV was significantly higher in intervention locations.

**Table 3.** HBCT impact on composition of individuals who have ever tested.

Panel A: Percent who have ever tested, by individual characteristics						
Subgroup	Control (%)	Intervention (%)	Delta	N	SE	p-value
Full sample	64.3	95.3	0.31	3343	0.01	0.00^***^
Males	53.7	92.7	0.39	1282	0.02	0.00^***^
Females	71.0	97.1	0.26	2031	0.01	0.00^***^
Age 15–25	89.8	97.6	0.08	261	0.03	0.01^***^
Age 25–35	82.5	97.7	0.15	900	0.02	0.00^***^
Age 35–45	69.2	94.4	0.25	720	0.03	0.00^***^
Age 45–60	53.5	93.0	0.40	829	0.03	0.00^***^
Age 60 and Over	35.8	95.1	0.59	629	0.03	0.00^***^

Panel B: Characteristics of individuals who have ever tested by testing source						
Characteristic	Tested due to HBCT	Already tested	Delta	N	SE	p-value
Male	43.5%	24.4%	− 0.19	2294	0.02	0.00^***^
Age	49.15	38.68	− 10.48	2271	0.63	0.00^***^
Ever married	93.0%	92.0%	− 0.01	2294	0.01	0.39
Attended school	81.0%	92.1%	0.11	2273	0.01	0.00^***^
Completed primary	58.4%	60.0%	0.02	2294	0.02	0.45
Years of education	8.11	8.99	0.87	1996	0.15	0.00^***^

Notes: ^***^p<0.01, ** *p*<0.05, *p<0.1.

### Impact of HBCT on individual beliefs and stigma

HBCT appears to have only slightly influenced individuals’ beliefs about prevalence of HIV and discordance in HIV status among couples. As shown in Table 4 (Panel A), in intervention locations respondents reported a lower estimate of the percent of couples in which at least one person has HIV (42.3% compared to 44.6%). Both of these numbers are significant overestimates of the true HIV prevalence in the community, which was around 5% in the 2003 Kenya Demographic and Health Survey (Central Bureau of Statistics, 2004), but HBCT appears to have reduced the beliefs about HIV prevalence slightly. However, the intervention also appears to have raised beliefs about how likely it is the partner of an HIV-positive person would also be HIV-positive (in intervention locations, respondents estimated that 75.4% of HIV-positive individuals have HIV-positive partners, compared to 73.9% in control locations). Panel B of Table 4 shows that HBCT did not significantly alter respondents’ beliefs about the importance of condom use.

**Table 4.** HBCT impact on individual beliefs.

Beliefs about prevalence and discordance	Control (%)	Intervention (%)	Delta	N	SE	p-value
% partners where one is +	44.6	42.3	− 0.02	3263	0.86	0.01^**^
If self +,% partner also +	73.9	75.4	0.02	3301	0.87	0.07^*^
If partner +,% also +	75.2	76.2	0.01	3299	0.85	0.24

Should use condom with …	Control	Intervention	Delta	N	SE	p-value
Spouse	2.35	2.37	0.02	3166	0.04	0.63
Regular partner	3.29	3.34	0.05	3173	0.04	0.17
Casual partner	3.83	3.84	0.01	3175	0.02	0.66
Sex worker	3.86	3.87	0.00	3173	0.02	0.88

Notes: For beliefs, these are respondents’ assessments of the likelihood on a 1–10 scale, which was then translated into percentage terms. For condom use, respondents were asked to agree or disagree, with 1 representing strong disagreement and 4 representing strong agreement;

*** *p*<0.01,

** *p*<0.05,

* *p*<0.1.

HBCT did appear to have an effect on stigma and hypothetical discrimination reported by community members (Table 5), but the effects were mixed. While the intervention led to a decrease in the sense that HIV was a sign of immoral behavior,^9^ both among respondents themselves and in the respondents’ perception of the community, it also increased the feeling of anger and disgust toward those with HIV. However, these responses were very variable, and when summarized in the stigma indices, only a small increase in self-stigma was significant. Moreover, the magnitude of the observed changes was small. HBCT also did not appear to alter opinions on appropriate behaviors toward individuals with HIV among household respondents.

**Table 5.** HBCT impact on individual stigma.

Panel A: All stigma measures						
	Control	Intervention	Delta	N	SE	p-value
Most people think HIV immoral	3.72	3.60	− 0.12	3302	0.05	0.02^**^
Self thinks HIV immoral	3.47	3.32	− 0.15	3321	0.06	0.01^***^
Would be ashamed if +	2.70	2.74	0.04	3321	0.05	0.52
Most are angry at those with HIV	2.93	3.05	0.12	3251	0.05	0.02^**^
Would be angry at self if +	2.65	2.75	0.10	3317	0.05	0.07^*^
People with HIV should be ashamed	2.31	2.38	0.07	3318	0.05	0.17
People with HIV should be blamed	2.39	2.40	0.00	3326	0.05	0.94
Most people feel disgust	2.86	3.01	0.15	3270	0.05	0.00^***^
Would be disgusted with self	2.47	2.56	0.08	3313	0.05	0.11
Most would think HIV+ teacher OK	1.95	1.98	0.03	3297	0.05	0.52
Self thinks HIV + teacher OK	1.78	1.79	0.01	3323	0.05	0.89
Most would not purchase from + vendor	2.20	2.16	− 0.03	3316	0.05	0.49
Self would not purchase from + vendor	1.99	1.89	− 0.09	3326	0.05	0.06^*^
Most think + govt official bad	1.86	1.86	0.00	3320	0.05	0.95
Most think + religious leader bad	2.21	2.22	0.01	3319	0.05	0.82
Self would not care for + person	1.38	1.38	0.00	3331	0.03	0.92
Would want + relative to be secret	2.66	2.69	0.03	3325	0.06	0.62

Panel B: Summary indices of stigma						
	Control	Intervention	Delta	N	SE	p-value
Stigma of most people toward HIV +	3.17	3.22	0.05	3175	0.04	0.20
Personal stigma toward HIV +	2.73	2.70	− 0.03	3301	0.04	0.51
Self stigma if infected	2.61	2.68	0.07	3295	0.05	0.10
Personal actions toward HIV +	1.88	1.84	− 0.04	3317	0.04	0.30
Community's actions toward HIV +	2.05	2.05	0.01	3283	0.04	0.90

Notes: Respondents were asked to agree or disagree, with 1 representing strong disagreement and 4 representing strong agreement (some questions were recoded so that higher numbers represent more stigma);

*** *p*<0.01,

** *p*<0.05,

* *p*<0.1.

The three outcome measures (beliefs about prevalence, condom use, and stigma) were correlated in revealing ways. Those with higher stigma placed lower importance on condom use (perhaps because they believed their own sexual partners to be unlike the people they stigmatize) while also holding stronger beliefs about prevalence and concordance (perhaps because they were less well informed, or perhaps because high perceived prevalence resulted in greater feelings of stigma).

### Impact of HBCT on community leader beliefs

HBCT did not appear to significantly influence either community leaders’ beliefs about HIV prevalence and discordance in HIV status, or their beliefs about the importance of condom use (Table 6). Beliefs about the prevalence of discordant couples and the likelihood of partner infection were higher in the intervention locations, but the differences were not statistically significant (potentially due to the small sample size of community leader respondents).

**Table 6.** HBCT impact on community leader beliefs.

Beliefs about prevalence and discordance	Control (%)	Intervention (%)	Delta	N	SE	p-value
% partners where one is +	42.4	45.4	0.03	259	3.35	0.38
If self +,% partner also +	71.7	73.1	0.01	260	3.22	0.67
If partner +,% also +	71.9	74.3	0.02	260	3.14	0.44

Should use condom with …	Control mean	Treatment Mean	Delta	N	SE	p-value
Spouse	2.67	2.71	0.04	301	0.14	0.74
Regular partner	3.70	3.79	0.09	301	0.08	0.26
Casual partner	3.90	3.95	0.05	301	0.04	0.23
Sex worker	3.93	3.96	0.03	301	0.04	0.51

Notes: For beliefs, these are respondents’ assessments of the likelihood on a 1–10 scale, which was then translated into percentage terms. For condom use, respondents were asked to agree or disagree, with 1 representing strong disagreement and 4 representing strong agreement; ***p <0.01, **p <0.05, *p <0.1.

Community leaders’ stigma toward individuals living with HIV was affected by HBCT in different ways from the household respondents (Table 7). Notably, HBCT altered community leaders’ opinions and their reported perceptions of others’ opinions on how HIV-positive individuals should be treated. Specifically, community leaders in HBCT areas were more likely to report that an HIV-positive teacher was acceptable, and that they themselves would buy food from an HIV-positive vendor. They also changed their reported perception of other people's beliefs in the same direction, and these changes were significant when summarized in indices for personal and perceived community beliefs about appropriate actions toward HIV-positive individuals.

**Table 7.** HBCT impact on community leader stigma.

Panel A: All stigma measures						
	Control	Intervention	Delta	N	SE	p-value
Most people think HIV immoral	3.58	3.53	− 0.05	301	0.20	0.80
Self thinks HIV immoral	3.43	2.99	− 0.44	301	0.20	0.03^**^
Would be ashamed if +	2.57	2.39	− 0.18	298	0.19	0.34
Most are angry at those with HIV	2.68	2.56	− 0.13	300	0.19	0.50
Would be angry at self if +	2.54	2.55	0.01	282	0.20	0.97
People with HIV should be ashamed	2.24	2.10	− 0.14	301	0.17	0.42
People with HIV should be blamed	2.01	2.07	0.05	301	0.17	0.76
Most people feel disgust	2.60	2.79	0.19	300	0.19	0.31
Would be disgusted with self	2.39	2.30	− 0.10	297	0.18	0.60
Most would think HIV+ teacher OK	2.05	1.42	− 0.64	300	0.15	0.00^***^
Self thinks HIV + teacher OK	1.68	1.37	− 0.30	301	0.13	0.02^**^
Most would not purchase from + vendor	1.89	1.62	− 0.26	301	0.14	0.06^*^
Self would not purchase from + vendor	1.69	1.42	− 0.27	301	0.13	0.03^**^
Most think + govt official bad	1.60	1.35	− 0.26	301	0.12	0.04^**^
Most think + religious leader bad	2.22	1.72	− 0.50	302	0.17	0.00^***^
Self would not care for + person	1.20	1.21	0.01	301	0.08	0.92
Would want + relative to be secret	2.50	2.77	0.27	301	0.20	0.19

Panel B: Summary indices of stigma						
	Control	Intervention	Delta	N	SE	p-value
Stigma of most people toward HIV +	2.95	2.96	0.00	300	0.14	0.99
Personal stigma toward HIV +	2.56	2.37	− 0.19	299	0.14	0.18
Self stigma if infected	2.49	2.40	− 0.08	280	0.16	0.61
Personal actions toward HIV +	1.67	1.40	− 0.28	300	0.11	0.01 ^**^
Community's actions toward HIV +	1.94	1.53	− 0.41	298	0.11	0.00 ^***^

Notes: Respondents were asked to agree or disagree, with 1 representing strong disagreement and 4 representing strong agreement (some questions were recoded so that higher numbers represent more stigma);

*** *p*<0.01,

** *p*<0.05,

* *p*<0.1.

## Discussion

This paper provides insight into the intersection between community leader opinions, community member opinions and characteristics, and a home-based HIV counseling and testing program in western Kenya. The results indicate that HBCT could be implemented in the presence of stigma and that the program was successful at reaching those types of individuals who are least likely to test on their own. Testing rates were actually higher in communities reporting higher stigma (perhaps due to the fact that HBCT reaches people who had previously declined to test, and individuals from high-stigma communities are less likely to have a previous test, or perhaps due to unobservable factors). This fact is consistent with the possibility that stigma deters people from testing because they do not want to see themselves or be seen as uniquely at risk for HIV, either due to their own stigmatized view or fear of stigma from others. When HIV testing is offered to an entire community, on the other hand, it removes this “singling out” effect. Policymakers should consider using “whole community” approaches to blunt the impact of stigma on HIV testing uptake.

We also found that HBCT had a powerful effect on community leader stigma beliefs, while having a more mixed impact on community member stigma. For community members, the testing increased feelings of anger toward HIV-positive individuals, but lowered the sense that having HIV was a sign of immoral behavior. The negative reaction may be because the intervention, in drawing greater attention to the fact that people with HIV are among community members, brings to the fore strong feelings about HIV one way or the other. It should also be noted that in this intervention, community members neither received a formal sensitization on HIV stigma, nor any educational initiative aside from the personal HIV counseling.

For community leaders, who were targeted for sensitization and education as part of the intervention, our results show an increased willingness to accept interaction with HIV-positive individuals (e.g., as a teacher or a vendor) and also a reduction in the view that HIV is associated with immorality. Community leaders also seem to indicate that the community shares their changing beliefs in accepting others (but not with respect to morality), but our results from the community members themselves do not support this.

The fact that the leaders’ beliefs about appropriate actions appear to change while their more deeply held beliefs about HIV (whether it should be a source of shame, whether they personally would feel shame, disgust, or anger with themselves if infected) do not change is telling about just how much impact is possible through community sensitization meetings. While standards of appropriate behavior (e.g., discrimination) appear to be possible to influence in a short period of time, more deeply held prejudices and values may only adjust over the course of a longer intervention, or even lived experience.

Our results indicate that HBCT cost-effectiveness may go beyond the dollars per individual tested, as this large-scale community intervention appears to produce ancillary impacts. First of all, it can reach people who may not otherwise put themselves forward for testing due to stigma. Secondly, if the power of HBCT can be harnessed to focus more on changing community members’ beliefs, knowledge, and practices simultaneously with testing, HBCT could deliver far more than scattered VCT interventions. But, this potential may need to be consciously deployed. Community leaders, who received an HIV sensitization, exhibited reduced stigma, while community members in this intervention were not targeted for sensitization, and exhibited more mixed effects. To maximize impact, future community testing interventions could include accompanying educational interventions directly targeting stigma.^10^

## Notes

5. Random sampling performed using Stata version 10.0 (StataCorp).

8. The final completion is lower than the target number due to some community leaders being unavailable during the survey period.

9. Our survey question did not define immoral behavior, but in Kenya the widely understood interpretation is infidelity. A minority of people may also interpret this as referring to intravenous drug use, or even witchcraft.

10. For examples of such interventions, see (Brown, Macintyre, & Trujillo, 2003).

## Appendix 1

**Stigma indices**:

*Community stigma*: Respondent's perception of the feelings of most people they know toward individuals with HIV.

Personal stigma: Respondent's own feelings toward individuals with HIV.

*Self stigma*: Respondents’ feelings toward being HIV positive themselves, either as a hypothetical or actual question.

*Personal stigma actions*: How an individual would treat a person with HIV, especially in the workplace.

*Community stigma actions*: How respondents believe most people they know would treat a person with HIV, especially in the workplace.^11^

**Appendix Table 1.** Drivers of community leader stigma beliefs.

Panel A: Multivariate regression of stigma on leader characteristics					
	Community stigma	Personal stigma	Self stigma	Personal actions	Community actions
Completed primary	− 0.0848	− 0.272	0.193	− 0.437^***^	− 0.350^**^
	(0.174)	(0.179)	(0.196)	(0.139)	(0.137)
Ever married	− 0.00206	0.411^*^	0.360	− 0.0780	− 0.366^**^
	(0.226)	(0.236)	(0.378)	(0.184)	(0.179)
Age	0.00556	0.0164^***^	0.00625	0.00573	0.00129
	(0.00609)	(0.00630)	(0.00682)	(0.00491)	(0.00468)
Male	0.384^**^	0.537^***^	0.318	0.272^*^	0.268^*^
	(0.180)	(0.185)	(0.209)	(0.147)	(0.143)
Evangelical	− 0.301^**^	− 0.284^**^	− 0.0903	− 0.145	0.0614
	(0.136)	(0.141)	(0.159)	(0.111)	(0.108)
Catholic	0.397^**^	0.494^***^	0.128	0.282^**^	0.110
	(0.167)	(0.171)	(0.193)	(0.135)	(0.132)
Anglican	0.115	0.0485	0.151	− 0.0836	− 0.137
	(0.157)	(0.164)	(0.185)	(0.128)	(0.124)

Panel B: Average stigma by leader religions						
	*N*	Comm stigma	Pers. stigma	Self stigma	Pers actions	Comm actions
Pentacostal	20	3.33	2.90	2.78	1.48	1.68
Catholic	64	3.28	2.86	2.55	1.77	1.82
Anglican	77	3.05	2.51	2.56	1.48	1.63
African Inland	33	3.03	2.65	2.77	1.76	2.02
SDA	16	2.48	2.10	1.77	1.28	1.47
Salvation Army	22	2.29	1.77	1.76	1.23	1.52

Note: Standard errors in parentheses;

*** *p*<0.01,

** *p*<0.05,

* *p*<0.1.

In Appendix Table 1, we examine the underlying drivers of community leader beliefs, to see which community leaders are likely to have greater stigma. We use bivariate regressions of each stigma measure on each underlying characteristic. The results are informative and they seem to suggest that higher education is associated with less stigma as self-reported by the community leaders. At the same time, men and older people have more stigma, while married individuals display more stigma toward people that they know. Of note is the fact that evangelical Christian groups appear to have lower levels of stigma than either Catholics or those who classify themselves as Anglican.

Panel B breaks down the religion results further, presenting simple averages of stigma by religion. With more fine distinctions, Pentacostal leaders express similar levels of stigma to Catholic leaders, but other evangelical groups such as the Salvation Army report much lower stigma.
